# Frailty and Oral Frailty: Conceptual Evolution, Evidence of Association and Progress in Intervention Research

**DOI:** 10.3390/nu18213671

**Published:** 2026-11-05

**Authors:** Wenfang Zhang, Hongkun Wu, Jin Feng

**Affiliations:** State Key Laboratory of Oral Diseases & National Center for Stomatology & National Clinical Research Center for Oral Diseases, Department of Geriatric Dentistry, West China Hospital of Stomatology, Sichuan University, Chengdu 610041, China; zhangwenfang1@stu.scu.edu.cn

**Keywords:** oral frailty, frailty, gerodontology, oral hypofunction, malnutrition, older adults

## Abstract

With the accelerating aging of the population, frailty has become a major geriatric syndrome affecting the health outcomes of older adults. Oral frailty—an early, reversible state in which oral function progresses from relative health to functional decline—has garnered attention in the fields of geriatric dentistry and geriatrics in recent years. Existing research suggests that oral frailty is associated with adverse health outcomes such as frailty, sarcopenia, malnutrition, disability, and mortality, and may hold value for early risk identification. However, at this stage, there are no unified standards for the definitions, assessment methods, or diagnostic criteria of frailty and oral frailty. Furthermore, oral frailty and systemic frailty appear to have a multifactorial and potentially bidirectional relationship, with mechanisms involving multiple pathways such as inadequate nutritional intake, decline in oral and systemic muscle function, chronic inflammation and changes in the oral microbiome, psychosocial withdrawal, and cognitive decline. Nevertheless, current evidence remains predominantly observational, and the causal relationship between these conditions—as well as whether oral interventions can improve long-term outcomes—still requires validation. Accordingly, this narrative review synthesizes the conceptual boundaries of oral frailty and its associated conditions, summarizes existing evidence from observational and intervention studies, and explores in depth the potential mechanisms underlying their association. Further research should evaluate the clinical utility of oral frailty screening within comprehensive geriatric assessment and determine whether interventions targeting oral frailty can translate improvements in oral function into clinically meaningful systemic and patient-important outcomes.

## 1. Introduction

As population aging accelerates, delaying functional decline and reducing the risks of disability and mortality have become major goals of healthy aging research and practice. The World Health Organization (WHO) defines healthy aging as “the process of developing and maintaining the functional ability that enables well-being in older age” [1]. Frailty is a key risk state during age-related functional decline. It is characterized by reduced physiological reserve, increased vulnerability, poor recovery from stressors, and a higher risk of adverse outcomes. Frailty is also potentially reversible [2,3,4]. Oral frailty was first proposed in the field of geriatric dentistry in Japan. It refers to an early and potentially reversible stage in the transition from relatively preserved oral function to clear oral functional decline [5,6]. Current evidence suggests that oral frailty is associated with frailty, sarcopenia, malnutrition, disability, and mortality. However, its causal relationship with these outcomes and the effects of intervention remain unclear. Although oral frailty has received increasing attention, significant uncertainties remain regarding its conceptual definition, assessment methods, relationship with systemic frailty, underlying mechanisms, and clinical implications. Previous reviews have summarized the prevalence, adverse outcomes, and interventions related to oral frailty [7,8,9], while more recent work has focused on oral rehabilitation, multidisciplinary management, and intervention strategies [10,11]. However, an important gap remains regarding the strength of evidence linking oral frailty to systemic frailty and the extent to which this association translates into clinical relevance. To address this gap, this review examines evidence from cross-sectional, longitudinal, and intervention studies separately, while considering differences in definitions and assessment tools as important sources of heterogeneity. We further evaluate whether improvements in oral function are accompanied by improvements in frailty or other patient-important outcomes. Accordingly, this review aims to clarify the concept and assessment of oral frailty and, more importantly, to explore whether it can move beyond risk identification to become a clinically actionable target for intervention.

## 2. Literature Search and Scope

We identified relevant English-language literature published through August 2026 using targeted searches of PubMed, Scopus, and Web of Science, and consulted ClinicalTrials.gov for relevant intervention studies. The search was updated during manuscript revision. Only English-language publications were included. Core search concepts included “oral frailty,” “oral dysfunction,” “oral hypofunction,” “frailty,” “sarcopenia,” and “dysphagia.” Representative search combinations included “oral frailty” AND “sarcopenia”; (“oral function” OR “oral dysfunction”) AND (“frailty” OR “sarcopenia”); and (“oral frailty” OR “oral dysfunction”) AND (“nutrition” OR “malnutrition” OR “dietary diversity”). Depending on the focus of each section, search terms were further expanded to include oral health and specific oral-function indicators, nutrition, inflammation, psychosocial and cognitive factors, and relevant interventions. Studies were selected according to their relevance to the concepts and assessment of oral frailty, related health outcomes, potential mechanisms, and oral or nutritional interventions. Earlier seminal publications, guidelines, and background literature were also included when they contributed to clarification of relevant concepts, mechanisms, or principles of clinical management. Nutrition-related studies were primarily identified by combining nutrition-related terms with oral frailty or oral-function concepts and by backward screening of the reference lists of relevant publications.

Findings were synthesized narratively and interpreted according to study design, study population, the definition and assessment of oral frailty, and outcome measures. When findings differed, variation in design, measurement methods, and study populations was considered rather than forcing a single conclusion. Observational studies were interpreted as evidence of association or prediction rather than causality. Intervention studies were assessed with particular attention to comparator design, follow-up duration, and whether outcomes were oral or systemic. No formal risk-of-bias assessment or evidence-quality grading was performed.

## 3. Conceptual Evolution of Frailty and Oral Frailty

### 3.1. Definition and Core Concepts of Frailty

There is currently no universally accepted definition of frailty. However, conceptual analyses and systematic reviews show substantial agreement regarding its core features. Frailty is generally characterized by an age-related decline in physiological reserve and function across multiple systems. This decline reduces the ability to cope with stressors and increases the risk of adverse outcomes, including disability, hospitalization, and mortality. Similarly, the World Health Organization (WHO) defines frailty as “a clinically identifiable condition among older adults associated with a decline in physiological reserve and function across multiple age-related organ systems, which may lead to impaired ability to cope with daily or acute stressors” [12].

In recent years, frailty has increasingly been recognized as a biopsychosocial geriatric syndrome rather than a purely physical condition [13,14]. It does not result from deterioration of a single organ system. Instead, it reflects the combined effects of reduced multisystem reserve, increased vulnerability, and impaired functional integration [2]. Accordingly, identifying, preventing, and managing it requires multidisciplinary collaboration among geriatrics, nursing, rehabilitation, nutrition, dentistry, and community health services.

Frailty should also be distinguished from normal aging, multimorbidity, and disability. It is neither an inevitable consequence of aging nor synonymous with multimorbidity or disability. Rather, it represents an important risk state between relatively preserved health and overt functional impairment. Although these conditions are conceptually distinct, they are closely related and may overlap. Both frailty and multimorbidity can predict disability. Disability may, in turn, increase the risk of frailty and multimorbidity, while multimorbidity may further accelerate the progression of frailty and disability [3].

### 3.2. Definition and Core Concepts of Oral Frailty

The concept of oral frailty originated in Japan. It is based on the view that oral function in older adults does not deteriorate abruptly. Instead, it declines gradually along a continuum from mild functional changes to overt impairment. Early identification and timely intervention may therefore help delay further deterioration of oral function [5]. In 2024, the Japan Geriatrics Society, the Japanese Society of Gerodontology, and the Japanese Association on Sarcopenia and Frailty issued a joint consensus statement on oral frailty. Oral frailty was defined as a potentially reversible state between healthy oral function and established oral hypofunction. The Oral Frailty 5-Item Checklist (OF-5) was also proposed as a simple screening tool for use in community and clinical settings [5].

Three related concepts require clear distinction: oral frailty, oral hypofunction, and oral diseases. Oral frailty refers to an early and mild decline in multiple aspects of oral function. It has relatively high reversibility potential and may serve as an early warning state for further functional deterioration [5,15]. In contrast, the Japanese Society of Geriatric Dentistry proposed oral hypofunction as a clinical diagnostic framework. It is diagnosed when abnormalities are present in at least three of seven domains: oral hygiene, oral dryness, occlusal force, tongue–lip motor function, tongue pressure, masticatory function, and swallowing function [6]. Oral hypofunction therefore represents a more established stage of functional impairment and may be less reversible than oral frailty. Oral diseases, such as dental caries, periodontitis, and tooth loss, primarily refer to specific pathological or structural conditions [16]. Although these three concepts are closely related, they are not interchangeable. They represent adjacent but distinct clinical constructs with different levels of functional impairment and different applications in screening, diagnosis, and management.

## 4. Assessment Tools for Frailty and Oral Frailty

### 4.1. Frailty Assessment Tools

No universally accepted gold standard exists for diagnosing frailty [17]. In both clinical practice and research, frailty is primarily assessed using validated screening tools, physical performance measures, and comprehensive clinical evaluation. Commonly used instruments include the Fried Frailty Phenotype (FFP), the Frailty Index (FI), the FRAIL scale, and the Clinical Frailty Scale (CFS) [4,18,19,20,21,22,23]. Table 1 provides a brief overview of commonly used methods for assessing frailty, along with their advantages and limitations.

Differences in the theoretical basis, assessment domains, and cutoff criteria of frailty instruments may introduce systematic variation in estimates of frailty prevalence and risk prediction [22]. For example, a meta-analysis of community-dwelling older adults in China reported prevalence estimates of 10.3%, 9.9%, and 10.8% using the FI, FFP, and FRAIL scale, respectively [23]. Although these differences were relatively modest, greater heterogeneity may arise across populations and clinical settings. Accordingly, interpretation and comparison of findings should account for the specific instrument, scoring system, and cutoff criteria employed. Estimates derived from different frailty instruments are not necessarily interchangeable because the tools capture partially distinct constructs and domains. When evidence from different instruments is synthesized, methodological heterogeneity should therefore be considered, with harmonization or calibration approaches applied where appropriate.

### 4.2. Oral Frailty Assessment Tools

As with frailty assessment, various approaches have been developed to assess oral frailty. These can be broadly grouped into three categories: self-reported screening instruments, objective oral-function measures, and multidimensional assessment tools. Among these, the Oral Frailty Index-8 (OFI-8) and the Oral Frailty 5-item Checklist (OF-5) are commonly used instruments [7,8,29,30], whereas the six-measure oral frailty assessment (often referred to as OFI-6) combines subjective and objective indicators for multidimensional assessment [31]. Individual objective tests can characterize specific functional domains but do not, by themselves, constitute oral frailty screening. Table 2 compares representative tools for oral frailty assessment and highlights their key strengths and limitations.

Because oral frailty instruments differ in their components, scoring systems, and cutoff criteria, populations classified as having oral frailty using different tools should not be regarded as directly equivalent [7,8]. Tool selection should reflect the purpose of assessment: the OFI-8 provides graded self-reported risk information, the OF-5 is suitable for rapid screening by non-dental professionals, and six-measure or other multidimensional approaches provide more detailed functional characterization and may be preferable when more comprehensive assessment is required. Studies should therefore report the instrument, scoring method, cutoff criteria, and assessment setting explicitly when presenting prevalence or risk estimates.

## 5. Evidence Linking Oral Frailty to Frailty

Current evidence on the relationship between oral frailty and frailty is derived mainly from cross-sectional studies, prospective cohort studies, systematic reviews, and intervention studies. The strength of inference varies across study designs. Cross-sectional studies mainly demonstrate associations and cannot establish temporal sequence or causality. Prospective cohort studies provide stronger evidence on temporal relationships and may indicate the predictive value of oral frailty for adverse health outcomes. Intervention studies are better suited to determining whether targeting oral frailty can improve functional or health outcomes. Therefore, in the following discussion of the evidence, we will not only examine whether oral frailty is associated with systemic frailty but also, by considering different study designs, analyze the evidential strength they provide regarding temporal relationships, independent effects, and potential causal inferences.

### 5.1. Evidence from Cross-Sectional Studies

Studies using composite definitions of oral frailty have reported significant associations between oral frailty and frailty, malnutrition, reduced dietary diversity, social withdrawal, and poorer quality of life among older adults [32,33,34,35,36]. These findings suggest that multidimensional oral frailty is associated with broader impairments in physical, nutritional, and social functioning. Existing research indicates that oral frailty often occurs in conjunction with a multidimensional cluster of physical functional decline, nutritional imbalance, and social functional impairment; however, this coexistence does not prove that oral frailty precedes these abnormalities chronologically, nor does it demonstrate that it independently drives these impairments.

Evidence based on individual oral structural or functional indicators should be considered separately. Systematic reviews and meta-analyses have shown that fewer remaining teeth, inadequate functional dentition, low tongue pressure, reduced occlusal force, impaired oral diadochokinesis, poor masticatory performance, and poor oral health status are associated with a higher prevalence of frailty [7,8,37,38]. A recent meta-analysis found that maximum occlusal force < 500 N was associated with both prefrailty and frailty [39]. In physically active older adults, lower bite force was also associated with oral frailty, although evidence directly comparing physically active and sedentary populations remains limited [40]. Although the aforementioned studies have confirmed a correlation between oral structural and functional impairments and frailty, this type of evidence cannot directly prove a link between oral frailty and frailty.

Cross-sectional findings should be interpreted with caution because oral frailty, oral hypofunction, and individual oral health or functional indicators are related but distinct constructs, and differences in their definitions and assessment methods may contribute to heterogeneity. Reverse causation remains possible, as frailty, sarcopenia, or malnutrition may themselves contribute to deterioration in oral function. Residual confounding by factors such as age, chronic diseases, polypharmacy, socioeconomic status, cognitive function, dietary habits, and access to healthcare may also influence the observed associations.

### 5.2. Evidence from Prospective Cohort Studies

Compared with cross-sectional studies, prospective cohort studies provide stronger evidence on temporal relationships. Using data from the Kashiwa Cohort, Tanaka et al. enrolled community-dwelling Japanese adults aged ≥65 years. At baseline, they assessed several oral health indicators, including number of teeth, masticatory ability, tongue pressure, and oral motor function. Participants were then followed for outcomes such as frailty, sarcopenia, disability, and mortality. Individuals with oral frailty at baseline had higher risks of these adverse outcomes during follow-up [31]. Subsequent studies using the OF-5 further showed that oral frailty, defined by five simplified indicators, was associated with increased risks of physical disability and mortality. These findings suggest that simplified screening tools may have value for risk prediction [30]. More recently, the longitudinal Frail Elderly in the Sasayama–Tamba Area study provided additional evidence outside the Kashiwa cohort, showing that baseline OF-5-defined oral frailty was associated with subsequent worsening of physical frailty over 2–3 years, although the longitudinal sample was smaller and sex-specific findings were not entirely consistent [41]. Among the longitudinal outcomes considered here, physical frailty is supported by findings from several cohorts, whereas evidence for sarcopenia is more limited; disability and mortality outcomes have been evaluated mainly within the Kashiwa cohort.

Overall, prospective cohort studies support oral frailty as an early risk marker for adverse health outcomes in older adults. Compared with cross-sectional studies, they provide stronger evidence that oral frailty may precede certain systemic outcomes. However, cohort studies remain observational. Residual confounding and reverse causation cannot be fully excluded, even after multivariable adjustment. For example, unrecognized sarcopenia, cognitive decline, or chronic inflammation may contribute to both oral functional decline and frailty progression. Conversely, frailty itself may also adversely affect oral function. Most longitudinal evidence comes from community-dwelling Japanese cohorts, where the concept of oral frailty and several commonly used assessment tools were developed. Instrument validation work has since expanded to China, Korea, and Singapore [42,43,44,45], but definitions, item composition, and cutoff values still differ across settings. Dietary patterns, access to dental care, oral health practices, socioeconomic conditions, and healthcare systems may further affect both prevalence and clinical meaning. Japanese cutoffs should therefore not be assumed to represent the same level of risk in other populations without local validation, and cross-national comparisons should account for both instrument choice and population-specific thresholds.

### 5.3. Evidence from Intervention Studies

Intervention studies are important for determining whether oral frailty is modifiable and whether improvements in oral function can lead to broader health benefits. Current evidence suggests that oral function training, swallowing therapy, dental treatment combined with nutritional support, and multidisciplinary management may improve proximal outcomes, including tongue pressure, oral motor function, masticatory performance, swallowing function, and oral health-related quality of life [9,10,46,47]. These intervention categories should not be considered equivalent. Exercise-based interventions have mainly been associated with improvements in oral motor function and tongue pressure, whereas swallowing therapy primarily targets swallowing function. Dental treatment may improve masticatory and oral health-related outcomes, while nutritional and multidisciplinary interventions may influence a broader range of oral, nutritional, and physical outcomes; however, the effects of such multicomponent programs cannot be attributed to the oral component alone. Takeuchi et al. [48] reported in a multi-arm randomized clinical trial that the primary outcome—the number of frailty criteria—decreased significantly over time across four groups receiving oral exercises at different frequencies. The study documents a temporal reduction in frailty criteria during oral-exercise programs, but it does not establish treatment efficacy relative to usual care or no intervention, because all four groups received oral exercise and no usual-care or non-exercise control group was included. Taken together, these findings suggest that some oral functional components related to oral frailty may be modifiable. However, most intervention studies have small sample sizes and short follow-up periods. Most outcomes remain focused on oral function, with limited evidence regarding systemic frailty outcomes. Evidence remains insufficient to determine whether these improvements translate into reduced frailty risk or other adverse systemic outcomes.

Overall, relatively consistent observational evidence supports an association between oral frailty and frailty. Prospective cohort studies further suggest that oral frailty may have prognostic value, whereas intervention studies mainly support short-term improvements in oral function, with only preliminary evidence regarding frailty outcomes. However, current evidence does not establish oral frailty as an independent cause of frailty. It also remains unclear whether oral interventions can delay or prevent frailty progression. Future studies should use standardized definitions and assessment tools for oral frailty and further clarify both the causal relationship between oral frailty and frailty and the clinical effectiveness of oral interventions. Table 3 summarizes representative prospective cohort studies, whereas key intervention studies and their oral versus systemic outcomes are summarized separately in Table 4.

## 6. Potential Mechanisms Linking Oral Frailty and Frailty

The relationship between oral frailty and frailty likely does not reflect a single linear causal pathway. Instead, it may arise from the interaction of multiple mechanisms, including inadequate nutritional intake, neuromuscular dysfunction, chronic inflammation and comorbidities, psychosocial factors, and cognitive decline.

### 6.1. Nutritional Pathway

Tooth loss, reduced occlusal force, chewing or swallowing difficulties, and xerostomia may limit the intake of foods such as meat, vegetables, fruits, and nuts. As a result, older adults may preferentially choose softer, less varied, and less nutrient-dense foods. This dietary shift may lead to inadequate intake of energy, protein, dietary fiber, and micronutrients [34,49,50]. Persistent nutritional insufficiency may contribute to weight loss, reduced muscle mass and strength, and fatigue, which may in turn increase vulnerability to frailty. Reduced dietary diversity may represent one potential nutritional link between oral frailty and physical frailty [34,51]. However, this relationship is likely to be bidirectional. Frailty, chronic diseases, functional limitations, and reduced appetite may also restrict dietary intake and further impair oral function.

Given this bidirectional relationship, nutritional assessment may be of value in clinical settings where the risk of malnutrition is high. In hospitalized older adults, nutritional screening may be complemented by selected laboratory indicators to improve the identification of malnutrition and nutritional risk [52].

Overall, oral functional decline, inadequate dietary intake, and systemic frailty may interact and reinforce one another over time, although the temporal sequence and relative contributions of these factors remain to be clarified.

### 6.2. Neuromuscular Pathway

The tongue, masticatory, and suprahyoid muscles play essential roles in chewing, swallowing, and speech. Impaired function of these muscles may reflect local muscle deterioration in the oral region or broader decline in systemic skeletal muscle function. Systematic reviews have shown that older adults with frailty or sarcopenia have lower tongue pressure, reduced occlusal force, impaired oral diadochokinesis, and poorer swallowing function. Dysphagia is also more prevalent in these populations [53]. These findings suggest that selected oral muscular impairments, including reduced tongue pressure and impaired oral motor or swallowing function, may partly reflect systemic sarcopenia. Objective measures of masticatory muscle structure may provide complementary information on oral neuromuscular status. In older adults with oral frailty, masticatory training has been shown to improve masseter muscle thickness together with maximum occlusal force and masticatory performance [54]. In addition, ultrasonographic masseter muscle thickness has been associated with handgrip strength, appendicular skeletal muscle mass, and calf circumference in older adults [55]. These findings suggest that structural and functional changes in the masticatory muscles may partly reflect broader skeletal muscle decline, although their causal relationship with systemic frailty remains uncertain. Conversely, these impairments may reduce food intake and thereby contribute to further nutritional and functional decline.

### 6.3. Inflammation, Chronic Disease, and Oral Microbiome Pathway

Oral frailty often coexists with periodontitis, poor oral hygiene, tooth loss, and xerostomia. These conditions may drive oral microbial dysbiosis and chronic local inflammation. Periodontitis, in particular, may promote systemic inflammation via the release of inflammatory mediators and translocation of oral bacteria or their products into the circulation. Frailty is similarly linked to chronic low-grade inflammation and immunosenescence [56,57,58], supporting a possible link between oral inflammatory burden and systemic inflammatory vulnerability. Nutritional status may also be relevant to this broader systemic environment. In hospitalized older adults, nutritional status has been associated with differences in adipokine and oxidative-stress profiles, including leptin, resistin, oxidized low-density lipoprotein, and glutathione peroxidase activity [59]. Although this evidence is not specific to oral frailty, it suggests that nutritional vulnerability may coexist with metabolic and oxidative abnormalities in older adults. The extent to which these alterations contribute to the association between oral frailty and systemic frailty remains uncertain.

### 6.4. Psychosocial and Cognitive Pathways

Oral health affects not only eating but also speech, self-confidence, social participation, and quality of life. Tooth loss, speech difficulties, xerostomia, halitosis, and embarrassment during eating may discourage older adults from dining out and engaging in social activities. Cross-sectional studies have found associations between oral frailty, social withdrawal, and poorer physical functioning [60,61], but the direction of these relationships is still uncertain. Evidence on depressive symptoms is more limited, and their role in this context remains unclear.

For cognitive decline, longitudinal evidence is available. Baseline oral frailty has been associated with subsequent mild cognitive impairment [62]. Cognitive impairment may also worsen oral hygiene, denture use, dietary choices, and treatment adherence, which could in turn affect oral health and function. However, longitudinal evidence for this reverse pathway is still limited. Overall, the available evidence supports an association between oral frailty and psychosocial or cognitive problems, but does not yet establish a reciprocal causal relationship.

Overall, the relationship between oral frailty and frailty is likely multifactorial and potentially bidirectional. However, the underlying causal pathways remain uncertain. Future studies should integrate longitudinal and interventional designs to clarify these mechanisms and determine whether improving oral function can lead to better frailty-related outcomes.

## 7. Advances in Interventions for Oral Frailty

### 7.1. Oral Function Training

Oral function training is one of the most extensively studied and feasible interventions for oral frailty. Common approaches include tongue exercises, lip and cheek exercises, tongue-pressure training, oral diadochokinesis exercises, articulation training, swallowing exercises, and orofacial muscle training. Systematic reviews suggest that these interventions can improve oral motor function, tongue pressure, tongue mobility, swallowing ability, and some aspects of masticatory function [9,46,47]. However, substantial heterogeneity exists in training content, frequency, duration, and supervision. Therefore, a standardized training protocol has not yet been established.

### 7.2. Dental Treatment and Prosthodontic Rehabilitation

Dental treatment and prosthodontic rehabilitation may help address oral conditions associated with functional decline in older adults. Tooth loss, inadequate functional dentition, poorly fitting dentures, reduced occlusal force, dental caries, periodontitis, and xerostomia can adversely affect masticatory efficiency, food selection, and nutritional intake. Observational studies have shown that fewer remaining teeth and inadequate functional dentition are associated with an increased risk of frailty [37,38,63]. Accordingly, clinical management may include treatment of dental caries and periodontitis, replacement of missing teeth when appropriate, denture adjustment, management of xerostomia, and maintenance of oral hygiene according to individual needs. These treatments are established components of dental care, but direct evidence that they improve the oral frailty construct or systemic frailty outcomes is limited.

### 7.3. Combined Oral and Nutritional Interventions

Oral frailty may impair food intake and dietary choice, while inadequate nutritional intake may further contribute to functional decline. In older adults with chewing difficulty, dysphagia, xerostomia, or nutritional risk, dietary counseling, adequate energy and protein intake, appropriate food-texture modification, and oral nutritional supplements when indicated may be considered according to individual needs [64,65]. These approaches are consistent with established geriatric nutrition practice, although their specific effects on oral frailty have not been clearly established.

Evidence for the added benefit of nutritional components in interventions relevant to oral frailty remains limited. In a cluster randomized controlled trial, Kito et al. compared physical exercise alone with a 12-week multicomponent program comprising physical exercise, oral exercises, brief dietary instruction, and textured lunch gatherings [66]. In this study, the textured lunches were intended to increase chewing demands by using harder foods, rather than to modify food consistency for dysphagia management. The multicomponent program improved tongue pressure and selected measures of physical function compared with physical exercise alone. However, because the oral, dietary, and physical components were delivered concurrently, the contribution of the nutritional component could not be separated from the contributions of the other intervention components. Other multicomponent interventions incorporating nutritional or dietary components have also reported improvements in oral frailty or related oral-function measures [11,47,67,68]. Taken together, these studies suggest potential benefit from multicomponent approaches, but heterogeneity in intervention content and the simultaneous delivery of several components make it difficult to determine whether nutritional support provides additional benefit beyond oral-function intervention alone. Whether such nutritional components improve systemic frailty also remains uncertain.

### 7.4. Multidisciplinary Integrated Interventions

Oral frailty often coexists with nutritional, swallowing, physical, and other functional impairments. Accordingly, several multicomponent interventions have combined oral-function training with nutritional support, physical activity, swallowing management, and other supportive measures [10,48,66,69]. These approaches have shown improvements in selected oral-function and physical-function outcomes; however, because multiple components are delivered concurrently, their independent contributions cannot be clearly determined, and evidence for effects on systemic frailty remains limited.

In clinical practice, multidisciplinary management may be tailored to individual needs, particularly when oral functional decline coexists with nutritional, swallowing, or physical impairments. It remains uncertain whether integrated programs confer benefits for systemic frailty, disability, or other patient-important outcomes; this question requires testing in larger comparative studies with extended follow-up. Representative intervention studies and their principal oral and systemic outcomes are summarized in Table 4.

**Table 4:** Representative intervention studies targeting oral frailty or oral functional decline and their oral versus systemic outcomes.

Study/Design	Population and Oral Frailty Assessment	Intervention/Comparator/Follow-Up	Oral-Function Outcomes	Systemic or Patient-Important Outcomes and Interpretation
Takeuchi et al., 2026 [48] Multi-arm randomized trial	Older adults with pre-frailty or frailty, Japan; *N* = 58. Participants were selected according to systemic frailty rather than oral frailty.	Same oral-exercise program at four frequencies; no usual-care or non-exercise control; 3 months	Number of oral-hypofunction criteria decreased over time (F = 16.456, *p* < 0.001, partial η^2^ = 0.234).	Frailty-criteria changes across the four groups: −0.36 (95% CI −0.79 to 0.07), −0.60 (−1.26 to 0.06), −0.57 (−1.38 to 0.24), and −0.60 (−1.01 to −0.19); overall time effect F = 14.803, *p* < 0.001, partial η^2^ = 0.215. Three of four CIs included zero. Because all groups exercised, efficacy versus no intervention cannot be established.
Kito et al., 2019 [66] Cluster RCT	Community-dwelling older adults, Japan; *N* = 86 (43/43). No composite oral-frailty definition; objective oral function was assessed.	Physical + oral exercise + dietary instruction + textured lunches vs. physical exercise alone; 12 weeks	Tongue pressure improved in the multicomponent group (*p* = 0.031).	Selected physical outcomes, including TUG, improved more favorably (*p* < 0.05), but frailty status, ADL and generic QoL were not established as improved. Oral-specific effects cannot be isolated from the dietary and physical components.
Tanaka et al., 2026 [68] Single-arm pre–post study	Community-dwelling adults ≥ 50 years from 18 Japanese regions; 651 enrolled, 518 paired analyses. Oral frailty assessed with OFI-8.	App-assisted CAMCAM-P; no concurrent control; 6 months	OFI-8: β = −0.29 (95% CI −0.51 to −0.16); oral-frailty risk: RR = 0.84 (95% CI 0.72–0.94).	KCL: β = −0.43 (95% CI −0.63 to −0.23); care-dependency-risk score: β = −0.33 (95% CI −0.56 to −0.10). These are relatively small changes in frailty/risk scores, not direct evidence of improved ADL or reduced incident care dependency; the uncontrolled design prevents causal attribution.

Abbreviations: ADL, activities of daily living; CI, confidence interval; KCL, Kihon Checklist; OFI-8, Oral Frailty Index-8; QoL, quality of life; RR, risk ratio; TUG, Timed Up and Go; CAMCAM-P, Comprehensive Awareness Modification of Mouth, Chewing, and Meal. Note: Oral-function outcomes are presented separately from systemic or patient-important outcomes because improvement in an oral physiological measure does not necessarily imply improvement in frailty, ADL, care dependency, or quality of life. Where 95% CIs were not reported in the original publication, they were not reconstructed from *p*-values. Statistical significance should not automatically be interpreted as clinical importance.

Intervention studies show that selected oral functional outcomes can improve, but evidence for systemic frailty or other patient-important outcomes remains insufficient. Whether oral frailty screening followed by targeted management improves clinically meaningful outcomes—and whether such an approach should become part of routine geriatric care—has not yet been established.

## 8. Discussion

Distinguishing oral frailty, systemic frailty, and related conditions, while interpreting the available evidence according to study design, is important not only for conceptual clarity but also for identifying clinically meaningful targets for intervention. Current evidence supports oral frailty mainly as a marker of increased vulnerability and a higher risk of frailty-related adverse outcomes. In contrast, evidence that oral frailty directly contributes to the development or progression of systemic frailty remains limited, and it is still unclear whether improvements in oral function translate into broader clinical benefits.

The next step is therefore to clarify the nature of the relationship between oral frailty and systemic frailty and to determine whether oral frailty itself, or specific components of it, represents a modifiable factor in frailty progression. Research should focus on identifying components that are not only associated with frailty but may also contribute to subsequent functional decline and are amenable to intervention. Longitudinal studies with repeated assessments are needed to establish temporal relationships more clearly, while well-controlled intervention studies should determine whether improvements in oral function translate into meaningful benefits in frailty, disability, and quality of life. Such evidence is needed to define the clinical value of oral frailty beyond risk identification and to determine whether it can serve as a practical target for frailty prevention and management.

## 9. Conclusions

Oral frailty may represent a potentially modifiable condition, and its early identification may provide opportunities for timely assessment and intervention. Current evidence suggests that oral frailty is associated with a range of adverse outcomes, including frailty, sarcopenia, malnutrition, reduced dietary diversity, social withdrawal, cognitive decline, disability, and mortality. The underlying mechanisms are likely multifactorial and may involve nutrition, neuromuscular dysfunction, inflammation and the oral microbiome, psychosocial factors, and cognitive function.

However, current evidence is insufficient to regard oral frailty as a single or independent cause of frailty. A more appropriate interpretation is that oral frailty may both reflect declining systemic functional reserve and contribute to further deterioration in nutritional, muscular, cognitive, and social function. Integrating oral health management into comprehensive frailty prevention and management may provide an additional approach to delaying functional decline, improving quality of life, and promoting healthy aging. Future research should move beyond confirming associations and focus on independent prognostic value, causal mechanisms, and intervention effectiveness. Particular attention should focus on identifying a clinically meaningful intervention window and determining which multidisciplinary strategies can improve long-term outcomes such as frailty, disability, and quality of life.

## Figures and Tables

**Table 1:** Comparison of Core Characteristics of Common Frailty Assessment Tools.

Assessment Tool	Items/Components	Criteria	Advantages	Limitations	Typical Applications
FFP [18,24]	5 items: weight loss, slow walking speed, reduced grip strength, low physical activity, and exhaustion	≥3 items: frail; 1–2 items: pre-frail; 0 items: robust	Highly standardized; strongest evidence for outcome prediction; good ability to identify pre-frailty	Limited to the physical domain; the original cohort excluded participants with neurological/ cognitive disorders	Epidemiological surveys; population stratification; screening in community and long-term care settings
FI [25]	≥30 health deficits (e.g., diseases, symptoms, signs, function, cognition, and psychological factors)	Continuous score (typically ≥0.25 indicates frailty)	Broadest multidimensional coverage; allows continuous quantification; good predictive performance	Large number of items; lower feasibility; no unified standard for deficit selection	Longitudinal database studies; assessment of overall health status
Modified Frailty Index-5 (mFI-5) [26,27]	5 items: diabetes, chronic obstructive pulmonary disease, congestive heart failure, hypertension requiring medication, and functional dependence	≥2 items: frail; <2 items: non-frail (some studies classify 1 item as pre-frail)	Very simple and practical; acceptable performance for predicting adverse postoperative outcomes	Markedly limited domains (no cognition, nutrition, or psychological status); cannot replace comprehensive geriatric assessment	Rapid perioperative screening; large surgical database studies
FRAIL Scale [21]	5 items: fatigue, resistance, ambulation, illnesses (≥5), and weight loss	≥3 items: frail; 1–2 items: pre-frail; 0 items: robust	Very concise; requires no equipment; suitable for primary care and large-scale initial screening	Self-reported and subjective; limited objectivity and granularity	Large-scale community screening; primary care settings
CFS [28]	9-point scale (1 = very fit; 9 = terminally ill)	≥5 indicates frailty (based on clinical judgment)	Highly intuitive; very rapid; applicable across a wide range of settings, including inpatient and emergency care	Substantial subjectivity; depends on assessor training and consistency; less sensitive to mild changes	Bedside assessment in inpatient/emergency settings; comprehensive geriatric assessment

Abbreviations: FFP, Fried Frailty Phenotype; FI, Frailty Index; mFI-5, Modified Frailty Index-5; CFS, Clinical Frailty Scale.

**Table 2:** Comparison of Representative Screening and Assessment Tools for Oral Frailty.

Assessment Tool	Items/ Components	Criteria	Advantages	Limitations	Typical Applications
OFI-8 [29]	8 items: difficulty chewing, choking, denture use, dry mouth, reduced frequency of going out, inability to chew hard foods, infrequent tooth brushing, and insufficient dental visits	The first 3 items are scored 2 points each and the remaining 5 items 1 point each (total score: 11). ≤2 = low risk; 3 = moderate risk; ≥4 = high risk.	Relatively comprehensive; the score has a clear quantitative association with oral frailty and the risk of functional impairment.	Entirely self-reported and lacks objective functional measures; results may be influenced by cognitive status and cultural background.	Initial screening of community-dwelling older adults; large-scale epidemiological surveys
OF-5 [5,30]	5 items: <20 remaining teeth, difficulty chewing, difficulty swallowing, dry mouth, and low articulatory oral motor skill	≥2 abnormal items = oral frailty	Very brief; easy to administer; suitable for rapid screening by non-dental professionals.	Further validation across diverse populations and settings is warranted; it provides less detailed functional information than multidimensional assessments incorporating objective measures.	Rapid community screening; primary care settings; non-dental specialty clinics
Tanaka six-measure oral frailty criteria (often referred to as OFI-6) [31]	6 components: number of teeth, masticatory performance, oral diadochokinesis (ODK; /ta/), tongue pressure, subjective difficulty eating tough foods, and subjective swallowing difficulty	≥3 impaired measures = oral frailty; 1–2 = pre-oral frailty	Integrates structural, objective functional, and subjective information; extensively used in research	Requires equipment and trained personnel; less feasible for rapid population screening	Detailed functional assessment; epidemiological and cohort studies

Abbreviations: OFI-8, Oral Frailty Index-8; OF-5, Oral Frailty 5-Item Checklist; ODK, oral diadochokinesis. Note: Oral hypofunction is not included in this table because it represents a distinct clinical diagnostic framework rather than an oral frailty screening instrument [6]. A positive oral frailty screening result should therefore not be interpreted as a diagnosis of oral hypofunction. For the OF-5, low articulatory oral motor skill can be assessed by self-reported difficulty with clear pronunciation in routine screening; in research settings, oral diadochokinesis (ODK) using repeated/ta/may be used for objective assessment, with <6.0 times/s indicating impairment [5,30]. Validation evidence: In Japanese community-dwelling cohorts, the OFI-8 (*n* = 2011) showed good discrimination for oral frailty (AUC 0.88, 95% CI 0.86–0.90; sensitivity and specificity 80% at ≥4), and each 1-point increase was associated with 1.3- and 1.1-fold higher risks of incident oral frailty and functional disability, respectively [29]. The OF-5 (*n* = 2031) showed predictive validity for incident physical frailty (adjusted HR 1.69, 95% CI 1.32–2.16), physical disability (1.40, 1.14–1.72), and mortality (1.44, 1.11–1.87) [30]. The six-measure criteria (*n* = 2011; ≥3 impaired measures) were associated with 2.4-, 2.2-, 2.3-, and 2.2-fold higher risks of physical frailty, sarcopenia, disability, and mortality, respectively [31].

**Table 3:** Representative prospective cohort studies examining associations between oral frailty and subsequent adverse health outcomes in older adults.

Evidence Type	Study	Population and Sample	Oral Frailty Definition/Intervention Target	Follow-Up or Intervention and Comparator	Main Outcomes and Effect Estimates	Interpretation and Key Limitations
Prospective cohort	Tanaka et al., 2018 [31]	Community-dwelling adults aged ≥65 years, Kashiwa, Japan; *N* = 2011	Six-domain oral frailty definition; ≥3 of 6 oral deficits	Physical frailty and sarcopenia assessed over 2 years; disability and mortality followed for approximately 45 months. Reference: oral non-frailty (0/6)	Oral frailty was associated with incident physical frailty: adjusted HR 2.41 (95% CI 1.27–4.55); sarcopenia: HR 2.13 (95% CI 1.05–4.58); disability: HR 2.35 (95% CI 1.18–4.67); mortality: HR 2.09 (95% CI 1.00–4.35).	Provides important early longitudinal evidence that accumulated oral deficits precede several adverse outcomes. However, the study is observational and cannot establish that oral frailty independently causes these outcomes; residual confounding and reverse causation remain possible.
Prospective cohort	Tanaka et al., 2023 [30]	Community-dwelling older adults without long-term care needs, Kashiwa, Japan; *N* = 2031; *n* = 1419 for incident physical frailty analysis	OF-5	Baseline 2012; repeated assessments through 2021; disability and mortality followed for 9 years. Reference: OF-5 score 0–1	Oral frailty was prospectively associated with incident physical frailty: adjusted HR 1.69 (95% CI 1.32–2.16); physical disability: HR 1.40 (95% CI 1.14–1.72); mortality: HR 1.44 (95% CI 1.11–1.87).	Supports the prognostic validity of the simplified OF-5. However, its incremental clinical utility beyond established geriatric assessments and its external validity outside Japanese community populations remain uncertain.
Prospective longitudinal cohort study	Kusunoki et al., 2025 [41]	Community-dwelling adults aged ≥65 years in the Sasayama–Tamba area, Hyogo, Japan; *N* = 934 at baseline and *N* = 329 in the longitudinal analysis	OF-5	2–3 years; observational follow-up with subsequent change in physical frailty assessed using the Japanese Cardiovascular Health Study (J-CHS) criteria	Baseline oral frailty was associated with subsequent worsening of J-CHS frailty status. In men, when oral frailty was defined dichotomously as OF-5 ≥ 2, it remained associated with frailty worsening (adjusted OR = 3.38, 95% CI 1.23–9.28; *p* = 0.018)	Provides longitudinal evidence from a cohort independent of the Kashiwa study, supporting an association between oral frailty and subsequent worsening of physical frailty. However, the longitudinal sample was relatively small (*N* = 329), baseline frailty prevalence was low, and sex-stratified analyses showed different patterns. In men, the association was significant when oral frailty was modeled dichotomously as OF-5 ≥ 2. The observational design does not establish causality.

Abbreviations: OF-5, Oral Frailty 5-Item Checklist; J-CHS, Japanese Cardiovascular Health Study criteria; HR, hazard ratio; OR, odds ratio; CI, confidence interval.

## Data Availability

No new data were created or analyzed in this study. Data sharing is not applicable to this article.

## Abbreviations

The following abbreviations are used in this manuscript

WHO	World Health Organization
FI	Frailty Index
CFS	Clinical Frailty Scale
FFP	Fried Frailty Phenotype
mFI-5	Modified Frailty Index-5
OFI-8	Oral Frailty Index-8
OF-5	Oral Frailty 5-item Checklist
J-CHS	Japanese Cardiovascular Health Study criteria
HR	Hazard ratio
OR	Odds ratio
CI	Confidence interval
AUC	Area under the curve
ADL	Activities of daily living
KCL	Kihon Checklist
QoL	Quality of life
RR	Risk ratio
TUG	Timed Up and Go
CAMCAM-P	Comprehensive Awareness Modification of Mouth, Chewing, and Meal
